# Supplementary material for: Effects of Pyrrole-Imidazole Polyamides Targeting Human TGF-β1 on the Malignant Phenotypes of Liver Cancer Cells
Source: Molecules. 2020 Jun 23;25(12):2883. doi: 10.3390/molecules25122883 (PMC7356887; doi:10.3390/molecules25122883)
Supplement: Supplementary file 1 [file molecules-25-02883-s001.pdf]

Supporting data

Title: Effects of pyrrole-imidazole polyamides targeting human TGF-β1 on the malignant phenotypes of liver cancer cells

By Keiko Takagi, *et al*

Contents;

Supplementary Figure S1

Supplemenraty Fig. S1

(a)

PI polyamide binding site

-1430 ACAGGAGGCTGCTTAGCCACATGGGAGGTGCTCAGTAAAGGAGAGCA ATTCTTAC AGGTGTCTGCCTCCT

FS2E

(b)

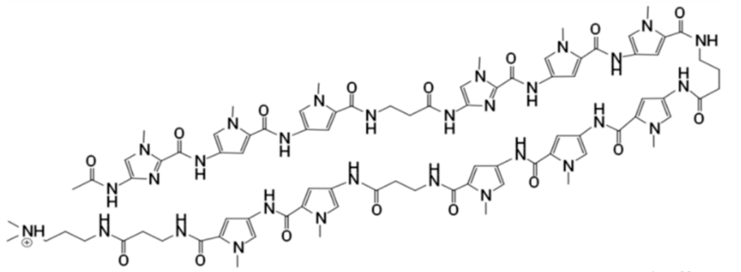

(c)

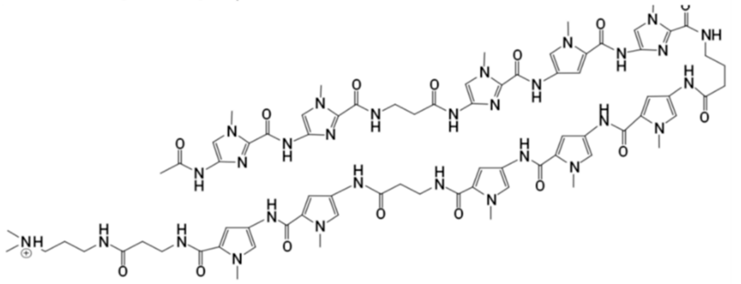

Supplementary Fig. S1. Target sequence of GB1101 on human *TGF-β1* promoter region and its structure. (Cited from ref 15 with permission)

(a) GB1101 was designed to bind to a DNA sequence near the FSE2 site (−1383 to −1376) upstream of the transcription initiation point in human *TGF-β1* promoter region (boxed). (b) Structure of GB1101 and (c) a mismatch PI polyamide.
